# Shannon Entropy of Gray Matter Eigenmodes: A Novel Biomarker for Alzheimer's Disease and Heterogeneous MCI Trajectories

**DOI:** 10.1002/advs.202511614

**Published:** 2025-11-04

**Authors:** Yumeng Li, Gaoping Long, Xinyue Zhang, Kewei Chen, Xin Li, Zhanjun Zhang

**Affiliations:** ^1^ Beijing Aging Brain Rejuvenation Initiative (BABRI) Centre Beijing Normal University Beijing 100875 China; ^2^ State Key Laboratory of Cognitive Neuroscience and Learning Beijing Normal University Beijing 100875 China; ^3^ College of Physics & Optoelectronic Engineering Jinan University Guangzhou 510632 China; ^4^ Banner Alzheimer's Institute Phoenix AZ 85006 USA

**Keywords:** Alzheimer's disease, amyloid‐β, biomarker, gray matter eigenmodes, structure‐function coupling

## Abstract

Current Alzheimer's disease (AD) diagnostics rely on late‐stage cognitive assessments or invasive biomarkers. Neuroimaging offers non‐invasive alternatives, but single‐modality approaches (structural atrophy or functional connectivity) face limitations in sensitivity and specificity for early detection. Entropy and temperature, novel structure‐function coupling (SFC) biomarkers based on gray matter eigenmodes, are introduced to quantify cortical disorganization in early AD. Using multimodal MRI and amyloid‐PET data from two cohorts (BABRI: N = 135; ADNI: N = 275), including cognitively normal (CN), mild cognitive impairment (MCI), and AD individuals, entropy is computed by projecting fMRI onto structural eigenmodes and temperature via eigenmode‐based functional connectivity reconstruction. These indices are tested for diagnostic classification, Aβ prediction, and MCI subtype stratification (reversed/stable/progressed). Entropy is significantly higher in AD than CN and MCI (Δ = 8–21%, *p* < 0.001) in both cohorts. Left‐hemisphere entropy yielded optimal diagnostic accuracy (AUC = 0.901 for CN vs MCI), while right/global entropy predicted Aβ burden (error reduction: 38.7–42.1%, *p* < 0.01). Entropy also distinguished MCI subtypes and captured biphasic changes in progressors. Temperature indices showed no significant group differences. Entropy from gray matter eigenmodes is a sensitive, non‐invasive biomarker for AD diagnosis and pathology prediction, revealing hemispheric asymmetries and nonlinear progression in MCI.

## Graphical abstract

Entropy derived from gray matter eigenmodes captures cortical disorganization in Alzheimer's disease. Using multimodal MRI and amyloid‐PET data, this novel structure–function coupling biomarker enables accurate classification of clinical stages, predicts Aβ burden, and stratifies mild cognitive impairment subtypes. The findings reveal hemispheric asymmetries and nonlinear progression, highlighting entropy as a sensitive, non‐invasive diagnostic tool.

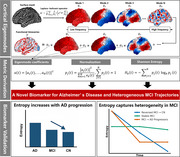

## 1 Introduction

Alzheimer's disease (AD), a highly prevalent neurodegenerative disorder, is known to begin its pathological progression 10–20 years before the onset of clinical symptoms.^[^ ^1^ ^]^ However, current diagnostic approaches primarily rely on late‐stage cognitive assessments and invasive cerebrospinal fluid (CSF) tests, which are limited by their invasiveness, high cost, and limited accessibility.^[^ ^2^ ^]^ Therefore, the development of non‐invasive, highly sensitive early screening techniques is crucial for identifying a precise intervention window for effective treatment of the disease.^[^ ^3^ ^]^

Neuroimaging has emerged as a promising non‐invasive biomarker for the early detection of Alzheimer's disease (AD) and cognitive impairment.^[^ ^4^ ^]^ Structural features such as hippocampal gray matter volume and medial temporal lobe atrophy have shown diagnostic value in imaging studies.^[^ ^5^, ^6^ ^]^ However, their practical application still faces several limitations. First, changes in individual gray matter volume and cortical thickness exhibit considerable inter‐individual variability.^[^ ^7^, ^8^ ^]^ These features are influenced by a range of genetic, environmental, and lifestyle factors. Although recent research advocates for multidimensional models that integrate genetic and environmental variables to assess early disease susceptibility, such comprehensive monitoring remains impractical for widespread use in the short term.^[^ ^9^ ^]^ Due to this variability and overlap, relying on a single gray matter feature may result in high false‐positive rates (misclassifying healthy individuals with naturally lower gray matter or faster aging as high risk) or false negatives (missing high‐risk individuals with early pathology but preserved gray matter).^[^ ^10^ ^]^ Differentiating between “normal aging” and “pathological” atrophy is particularly challenging during the early disease stages.^[^ ^11^, ^12^ ^]^ Second, in the earliest detectable phases of AD—such as preclinical AD or early mild cognitive impairment(MCI)—structural changes in gray matter may be subtle and focal.^[^ ^13^, ^14^ ^]^ These early alterations are often too minor to be reliably captured by structural MRI alone. As a result, using gray matter changes as early indicators may lead to missed intervention windows. Moreover, gray matter atrophy progresses relatively slowly, and a single time‐point measurement cannot capture the dynamic speed of disease progression.^[^ ^15^, ^16^ ^]^ Resting‐state functional MRI (rs‐fMRI), another key neuroimaging modality, can detect brain network dysfunction, which may precede structural atrophy. It has shown great potential for early screening of individuals at risk for AD and MCI.^[^ ^17^, ^18^, ^19^ ^]^ However, like structural markers, functional connectivity also suffers from high variability and temporal lag when used as a single diagnostic feature.^[^ ^20^ ^]^ Additionally, brain network data are extremely high‐dimensional, involving thousands of connections.^[^ ^21^ ^]^ Identifying disease‐relevant features requires a large number of statistical comparisons, increasing the risk of false positives. Furthermore, many functional connectivity alterations lack spatial specificity for AD and are difficult to replicate across studies due to methodological inconsistencies and limited standardization.^[^ ^22^ ^]^

While multi‐modal neuroimaging methods have been proposed to overcome the limitations of the single‐modal for early Alzheimer's disease (AD) detection.^[^ ^23^, ^24^, ^25^ ^]^ More specifically, these shortcomings can be explained from two perspectives. First, most studies rely on a simplistic “feature stacking” strategy, where structural features and functional connectivity are independently extracted and subsequently fused. Crucially, this post‐hoc combination fails to capture the inherent, dynamic interdependencies between brain structure and function that are likely central to pathological processes. Second, the dominant paradigm concentrates on modeling associations between white matter connectivity and resting‐state functional connectivity.^[^ ^26^, ^27^ ^]^ In fact, this white‐matter‐centric structure–function coupling(SFC) framework faces some key challenges for early AD screening, which can be listed as follows:

- White matter connectomes often show low accuracy in predicting functional connectivity patterns.^[^ ^28^ ^]^ Thus, this white‐matter‐centric SFC cannot provide a mechanism to explain the abnormal SFC in early AD. Essentially, there is considerable functional redundancy not constrained by the specific white matter pathways being measured.
- White matter connectivity primarily describes long‐range, inter‐regional communication pathways. Consequently, it overlooks the critical physical constraints imposed by gray matter microstructure and geometry at the local microcircuit level.^[^ ^29^, ^30^ ^]^ This omission is particularly detrimental, since accumulating evidence suggests that early AD pathology (e.g., molecular disruptions, astrocytic dysfunction) begins in the local gray matter microenvironment.^[^ ^31^, ^32^ ^]^ These changes occur well before widespread white matter degeneration or large‐scale network failure becomes apparent.^[^ ^33^ ^]^

The shortcomings of previous research on SFC suggest that we focus on the dynamic interactions between gray matter structure and brain function. A widely adopted approach in this direction is the eigenmode method, by which the eigenmodes determined by the structural information are used to analyze the brain function.^[^ ^34^, ^35^, ^36^ ^]^ Recent work published in Nature has demonstrated that eigenmodes derived from cortical geometry—not white matter connectivity—can effectively explain and predict large‐scale functional brain activity.^[^ ^30^ ^]^ This challenges the long‐standing paradigm that “white matter connectome determines function,” suggesting instead that the geometry of gray matter imposes a more fundamental constraint on functional organization. Moreover, early Alzheimer's disease (AD) pathology is thought to emerge within the gray‐matter microenvironment—e.g., astrocytic and ionic dysregulation—before large‐scale white‐matter degeneration becomes detectable.^[^ ^37^, ^38^, ^39^, ^40^ ^]^ If cortical geometry constrains neural dynamics, then the brain's intrinsic eigenmodes provide a natural basis in which to quantify how ongoing activity distributes across geometry‐defined modes. Therefore, we propose that gray matter eigenmodes, which serve as constraints on brain function, may capture subtle alterations associated with the progression of AD better.

Building upon this paradigm shift, we introduce two novel SFC indexes based on gray matter eigenmodes in this article. The first index is the entropy of the distribution of the brain function over the eigenmodes. Specifically, by projecting resting‐state fMRI time series onto the gray matter eigenmode basis to compute activation coefficients for each mode, the Shannon entropy of these coefficients quantifies the dispersion of functional activity across eigenmodes. In other words, lower entropy values indicate that activity is concentrated in fewer modes, which reflects a more ordered state; Higher entropy values indicate functional dispersion across a broader range of eigenmodes, which reflects a more disordered and distributed activation profile. The second index is the temperature of the reconstructed whole‐brain functional connectivity matrix based on the gray matter eigenmodes. Specifically, we assess the predictive accuracy of this reconstruction across eigenmodes ranked by frequency, and fit it to a cumulative distribution function of exponential decay distribution. Then, the temperature is given by the exponential decay coefficient. A steeper decay (lower temperature) suggests that low‐frequency modes dominate the functional architecture, while a flatter decay (higher temperature) indicates greater contributions from high‐frequency modes. Together, these indexes offer a principled and interpretable framework for quantifying the structure‐function relationship grounded in cortical geometry, thereby advancing a geometry‐informed model of brain dynamics.

In this study, we provide preliminary validation of entropy‐ and temperature‐based indexes for distinguishing between normal and pathological aging. We hypothesize that individuals with pathological aging will exhibit significantly greater disorder in brain system dynamics, and that these indexes offer enhanced sensitivity for detecting preclinical Alzheimer's disease (AD) along with more specific mechanistic insight. In particular, we aim to establish Shannon entropy as a novel, non‐invasive biomarker for tracking AD progression, given its responsiveness to cortical network disorganization beyond conventional structural or functional measures. To further address the heterogeneity of mild cognitive impairment (MCI), we stratify clinically defined MCI cases into reversed, stable, and progressed subtypes based on longitudinal entropy trajectories. This framework is applied across two multimodal cohorts (BABRI, N = 135; ADNI, N = 275), using entropy and temperature indexes to evaluate diagnostic specificity, characterize disease progression, and predict amyloid‐β (Aβ) burden, with machine learning and regression models optimized for classification and biomarker associations.

## 2 Results

### 2.1 Participant Characteristics

The clinical characteristics of participants from both the BABRI (N = 135) and ADNI (N = 275) cohorts revealed distinct demographic and cognitive profiles across diagnostic groups (See **Table** **1**). In the BABRI cohort, no significant differences were observed in sex distribution (χ^2^ = 3.32, *p* = 0.19), age (*F* = 0.51, *p* = 0.61), or education years (*F* = 1.95, *p* = 0.15) among groups, whereas marked cognitive stratification emerged in Mini‐Mental State Examination (*F* = 152.3, *p* < 0.001) and Auditory Verbal Learning Test (*F* = 48.5, *p* < 0.001). Similarly, the ADNI cohort showed nonsignificant sex (χ^2^ = 1.21, *p* = 0.55) and age differences (*F* = 2.14, *p* = 0.12), though education years exhibited marginal variation (*F* = 3.86, *p* = 0.02), with pronounced cognitive decline in MMSE (*F* = 236.2, *p* < 0.001) and AVLT (*F* = 98.8, *p* < 0.001). Both cohorts demonstrated progressive cognitive deterioration from NC to AD (all pairwise *p* < 0.05, Bonferroni‐corrected), underscoring diagnostic validity while reflecting cohort‐specific demographic influences. Given the significant between‐group differences in education levels within the ADNI cohort, all subsequent analyses utilized residualized variables adjusted for demographic confounders (age, sex, and education years) through linear regression modeling to mitigate potential confounding effects.

**Table 1:** Characteristics and neuropsychologic test results.

	Total	NC	MCI	AD	F/χ2	partial η^2^	*P*
	n = 135	n = 76	n = 30	n = 29			
**BABRI sample (N = 135)**							
Sex (female, %)	79 (58.5%)	43 (56.6%)	15 (50%)	21 (72.4%)	3.32	0.05	> 0.05 ^1,2,3^
Age (years)	68.23 ± 8.09	67.08 ± 7.63	68.73 ± 8.35	70.72 ± 8.65	0.51	0.01	> 0.05 ^1,2,3^
Education (years)	12.60 ± 3.80	13.19 ± 3.56	12.07 ± 3.35	11.62 ± 4.62	1.95	0.03	> 0.05 ^1,2,3^
Aβ accumulation	0.99±0.12	0.95±0.11	1.00±0.11	1.08±0.13	13.14	0.17	< 0.001 ^1,2,3^
MMSE	24.11 ± 6.96	27.58 ± 1.65	26.30 ± 1.56	12.76 ± 7.11	152.3	0.70	< 0.001 ^1,2,3^
AVLT	21.63 ± 12.20	29.08 ± 8.90	16.90 ± 8.15	6.46 ± 4.68	48.5	0.42	< 0.001 ^1,2,3^
**ADNI sample (N = 275)**	n = 275	n = 107	n = 126	n = 42			
Sex (female, %)	140 (50.9%)	59 (55.1%)	60 (48%)	21 (50%)	1.21	0.01	> 0.05 ^1,2,3^
Age (years)	72.53±6.99	72.27±6.55	72.06±7.30	74.56±6.95	2.14	0.02	> 0.05 ^1,2,3^
Education (years)	16.24±2.66	16.82±2.37	15.97±2.87	15.57±2.47	3.86	0.03	< 0.05 ^1,2,3^
Aβ accumulation	27.49±2.83	28.97±1.26	27.97±1.79	22.31±2.37	37.112	0.21	< 0.001 ^1,2,3^
MMSE	27.49±2.83	28.97±1.26	27.97±1.79	22.31±2.37	236.2	0.64	< 0.001 ^1,2,3^
AVLT	38.04±12.50	46.08±9.80	36.53±10.16	22.02±6.43	98.8	0.42	< 0.001 ^1,2,3^

*Note*: The measured data are represented by the mean and standard deviation. The p value for sex was obtained using a Chi‐square test. AVLT: Auditory Verbal Learning Test; *p* ^1^ indicates a significant difference between the NC and MCI groups. *p* ^2^ indicates a significant difference between the NC and AD groups. *p* ^3^ indicates a significant difference between the MCI and AD groups, *p* < 0.05, the same as below.

### 2.2 Entropy and Temperature Profiles across Diagnostic Groups

**BABRI Cohort (N = 135)**: Significant intergroup differences emerged in entropy measures but not in temperature indexes. For **entropy in left hemisphere**, ANCOVA revealed moderate group differences (F = 3.07, *p* < 0.05), with post hoc comparisons indicating AD exhibited higher entropy than both NC (*p* < 0.05) and MCI (*p* < 0.01). **Entropy in right hemisphere** demonstrated stronger discrimination (F = 7.64, *p* < 0.001), where AD > NC (*p* < 0.001) and AD > MCI (*p* < 0.01). Global entropy (**entropy**) showed intermediate effects (F = 4.1, *p* < 0.01), with AD exceeding NC (*p* < 0.05) and MCI (*p* < 0.01). In contrast, temperature parameters showed no significant group differences (all F < 13.14, *p* > 0.05), suggesting temperature indexes lack diagnostic specificity in this cohort.

**ADNI Cohort (N = 275)**: Entropy measures showed robust diagnostic stratification. **Entropy in left hemisphere** (F = 10.77, *p* < 0.001) differentiated AD from NC (*p* < 0.001) and MCI (*p* < 0.01). Similarly, **entropy in right hemisphere** (F = 12.08, *p* < 0.001) and global **entropy** (F = 12.21, *p* < 0.001) exhibited AD > NC (*p* < 0.001) and AD > MCI (*p* < 0.001). Temperature indexes again showed no group differences (all F < 0.49, i > 0.05), reinforcing their limited discriminative capacity.

**Figure 1:**
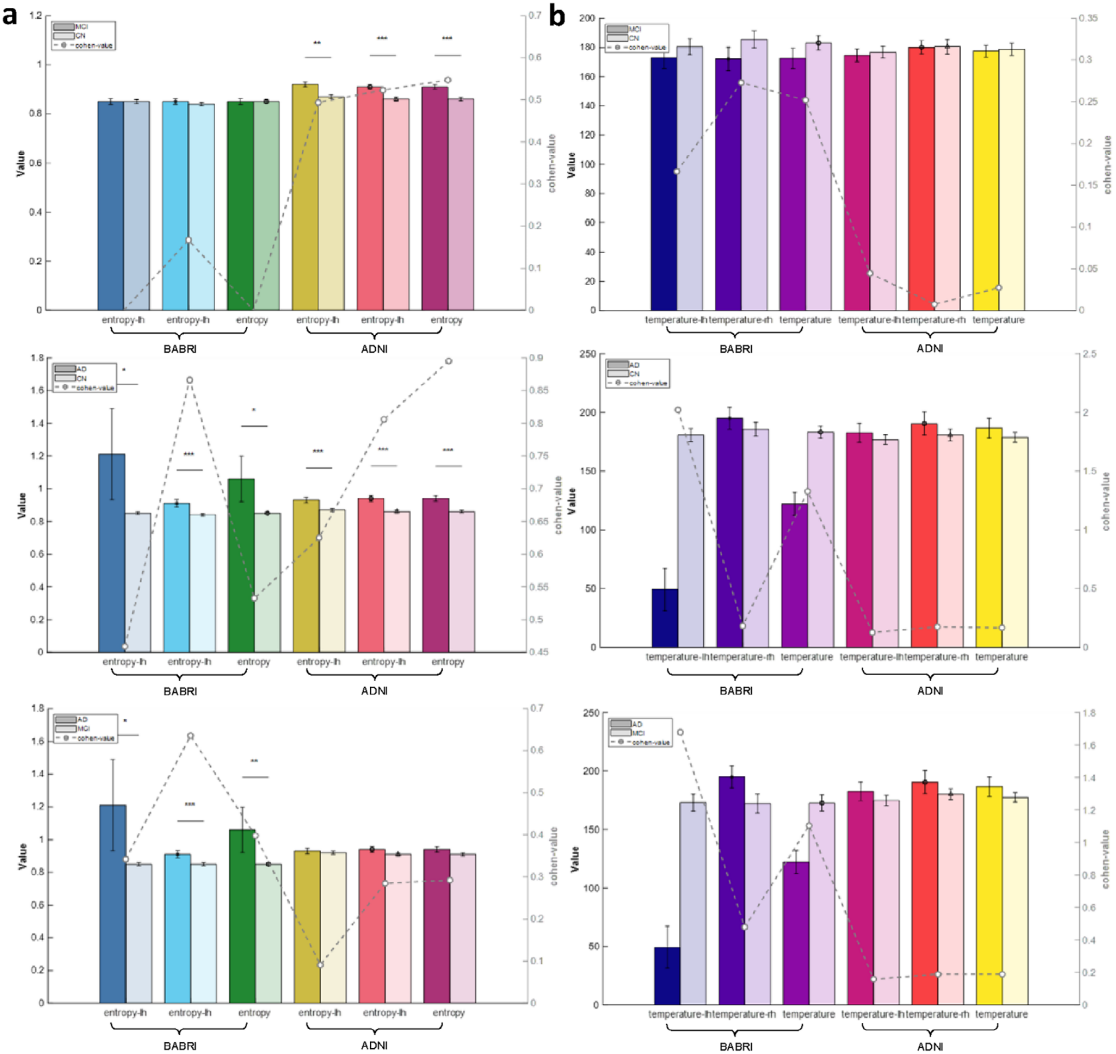
The inter‐group distribution and effect intensity of entropy and temperature indicators in diagnosis. a) Entropy is higher in AD than NC and MCI across left, right, and whole‐brain metrics (*p* < 0.05; ≈12–15% higher in AD). b) Temperature indexes show no significant intergroup differences in either cohort (all F < 0.5, *p* > 0.05). Sample sizes: BABRI cohort: total = 135 (NC = 76, MCI = 30, AD = 29); ADNI cohort: total = 275 (NC = 107, MCI = 126, AD = 42).

The visualization details of the above results are shown in **Table** **2** and **Figure** **1**. Both cohorts demonstrated that AD groups showed 12–15% higher entropy values than NC/MCI (BABRI: Δentropy = 0.21; ADNI: Δentropy = 0.08), with effect sizes (η^2^) ranging 0.18–0.31. Despite cohort differences in baseline values (BABRI temperature: 168.02 ± 164.83; ADNI: 179.36 ± 46.35), temperature indexes consistently failed to distinguish diagnostic groups. These results highlight entropy as a potential biomarker for AD‐related cortical disorganization, while temperature measures may reflect nonspecific age‐related changes.

**Table 2:** Group differences in Entropy and Temperature in BABRI and ADNI Cohorts.

	Total	NC	MCI	AD	F	partial η^2^	*P*
	n = 135	n = 76	n = 30	n = 29			
**BABRI sample (N = 135)**							
entropy_lh	0.93±0.69	0.85±0.07	0.85±0.06	1.21±1.50	3.07^*^	0.04	< 0.05 ^2,3^
entropy_rh	0.86±0.08	0.84±0.06	0.85±0.06	0.91±0.12	7.64^***^	0.10	< 0.01 ^2^; < 0.001 ^3^
entropy	0.89±0.35	0.85±0.06	0.85±0.06	1.06±0.75	4.10^**^	0.06	< 0.05 ^2^; < 0.01 ^3^
temperature_lh	151.45±320.95	180.59±47.88	172.96±40.07	49.28±696.84	1.82	0.03	> 0.05 ^1,2,3^
temperature_rh	184.60±49.82	185.66±51.05	172.21±44.40	195.03±50.83	0.77	0.01	> 0.05 ^1,2,3^
temperature	168.02±164.83	183.12±43.25	172.58±37.91	122.16±352.48	0.15	0.002	> 0.05 ^1,2,3^
**ADNI sample (N = 275)**	n = 275	n = 107	n = 126	n = 42			
entropy_lh	0.90±0.10	0.87±0.09	0.92±0.11	0.93±0.11	10.77^***^	0.14	< 0.01 ^1^; < 0.001 ^2^
entropy_rh	0.89±0.10	0.86±0.09	0.91±0.10	0.94±0.12	12.08^***^	0.16	< 0.001 ^1^; < 0.001 ^2^
entropy	0.90±0.10	0.86±0.08	0.91±0.10	0.94±0.11	12.21^***^	0.16	< 0.001 ^1^; < 0.001 ^2^
temperature_lh	176.75±47.20	176.80±43.01	174.73±49.04	182.62±52.29	0.41	0.01	> 0.05 ^1,2,3^
temperature_rh	181.98±53.68	180.64±52.45	180.25±50.98	190.52±64.22	0.45	0.01	> 0.05 ^1,2,3^
temperature	179.36±46.35	178.72±43.45	177.49±46.17	186.57±53.93	0.49	0.01	> 0.05 ^1,2,3^

*Note*: The measured data are represented by the mean and standard deviation. The suffix *_lh* indicates the entropy or temperature metric calculated for the **left hemisphere**; *_rh* indicates the **right hemisphere**; variables **without a suffix** denote the **whole‐brain** metric; *p* ^1^ indicates a significant difference between the NC and MCI groups. *p* ^2^ indicates a significant difference between the NC and AD groups. *p* ^3^ indicates a significant difference between the MCI and AD groups, *p* < 0.05.

### 2.3 Entropy Captures Heterogeneity in MCI Subtypes and Associates with Amyloid‐β Accumulation

Our findings revealed that differences between CN and MCI were only observed in the ADNI dataset, while distinctions between MCI and AD emerged exclusively in the BABRI dataset. This divergence may stem from the fact that MCI populations in ADNI and BABRI represent distinct transitional phases along the CN‐to‐AD continuum. Specifically, MCI individuals in ADNI might be closer to the AD conversion stage, whereas those in BABRI likely remain in a more stable compensatory phase. Based on this observation, we propose a sub‐hypothesis that the Shannon entropy of brain activity could capture the heterogeneity within clinically defined MCI states. To test this, we stratified the MCI cohort in the ADNI dataset (N = 275) into three subgroups: reversed MCI (transitioning to CN), stable MCI (remaining MCI), and progressed MCI (transitioning to AD) (**Table** **3**).

**Table 3:** Group Differences in Entropy and Temperature Across MCI Progression Subgroups in the BABRI and ADNI Cohorts.

ADNI sample	NC	MCI‐CN	MCI‐MCI	MCI‐AD	AD	F	*P*
(N = 275)	n = 107	n = 26	n = 64	n = 35	n = 43		
Aβ accumulation	1.11±0.15	1.05±0.11	1.20±0.23	1.35±0.21	1.43±0.22	31.85^***^	> 0.05 ^19^; < 0.001 ^2,3,4,5,6,7,8^
entropy_lh	0.87±0.09	0.91±0.11	0.92±0.11	0.92±0.10	0.93±0.11	5.24^***^	< 0.001 ^1,2,3,4^
entropy_rh	0.86±0.09	0.91±0.11	0.91±0.09	0.91±0.10	0.94±0.12	5.69^***^	< 0.001 ^1,2,3,4^
entropy	0.86±0.08	0.91±0.10	0.92±0.10	0.92±0.10	0.93±0.11	5.84^***^	< 0.001 ^1,2,3,4^
temperature_lh	176.80±43.01	183.46±58.50	175.30±46.69	167.21±45.79	182.28±51.71	0.75	> 0.05 ^1,2,3,4,5,6,7,8,9^
temperature_rh	180.64±52.45	190.48±63.06	178.97±50.45	175.01±41.53	192.64±64.96	0.92	> 0.05 ^1,2,3,4,5,6,7,8,9^
temperature	178.72±43.45	186.97±56.71	177.13±44.27	171.11±40.86	187.46±53.60	0.96	> 0.05 ^1,2,3,4,5,6,7,8,9^

Note: MCI‐CN: MCI at baseline, reverted to NC during follow‐up. MCI‐AD: MCI at baseline and progressed to AD until the last visit. MCI: MCI at baseline and maintained cognitive impairment. *p* ^1^ indicates a significant difference between the NC and MCI‐CN groups. *p* ^2^ indicates a significant difference between the NC and MCI groups. *p* ^3^ indicates a significant difference between the CN and MCI‐AD groups. *p* ^4^ indicates a significant difference between the CN and AD groups. *p* ^5^ indicates a significant difference between the MCI‐CN and MCI groups. *p* ^6^ indicates a significant difference between the MCI‐CN and MCI‐AD groups. *p* ^7^ indicates a significant difference between the MCI‐CN and AD groups. *p* ^8^ indicates a significant difference between the MCI and MCI‐AD groups. *p* ^9^ indicates a significant difference between the MCI and AD groups. *p* ^10^ indicates a significant difference between the MCI‐AD and AD groups.

Neurobiological characteristics exhibited significant heterogeneity across five cognitive transition groups (NC, MCI‐CN, MCI‐MCI, MCI‐AD, AD). Amyloid‐β (Aβ) accumulation demonstrated a clear pathological continuum with entropy indexes, whereas temperature parameters showed no significant group differences. Aβ levels progressively increased with disease severity (*F*  = 31.85, *p* < 0.001), with the NC group (1.05 ± 0.11) showing significantly lower values than the MCI group (1.20 ± 0.23, *p* < 0.001), MCI‐AD subgroup (1.35 ± 0.21, *p* < 0.001), and AD group (1.43 ± 0.22, *p* < 0.001). Notably, the MCI‐AD subgroup exhibited higher Aβ levels compared to both MCI‐CN (1.35 vs 1.05, *p* < 0.001) and MCI‐MCI subgroups (1.35 vs 1.20, *p* < 0.001), underscoring the critical role of Aβ accumulation in AD progression.

**Figure 2:**
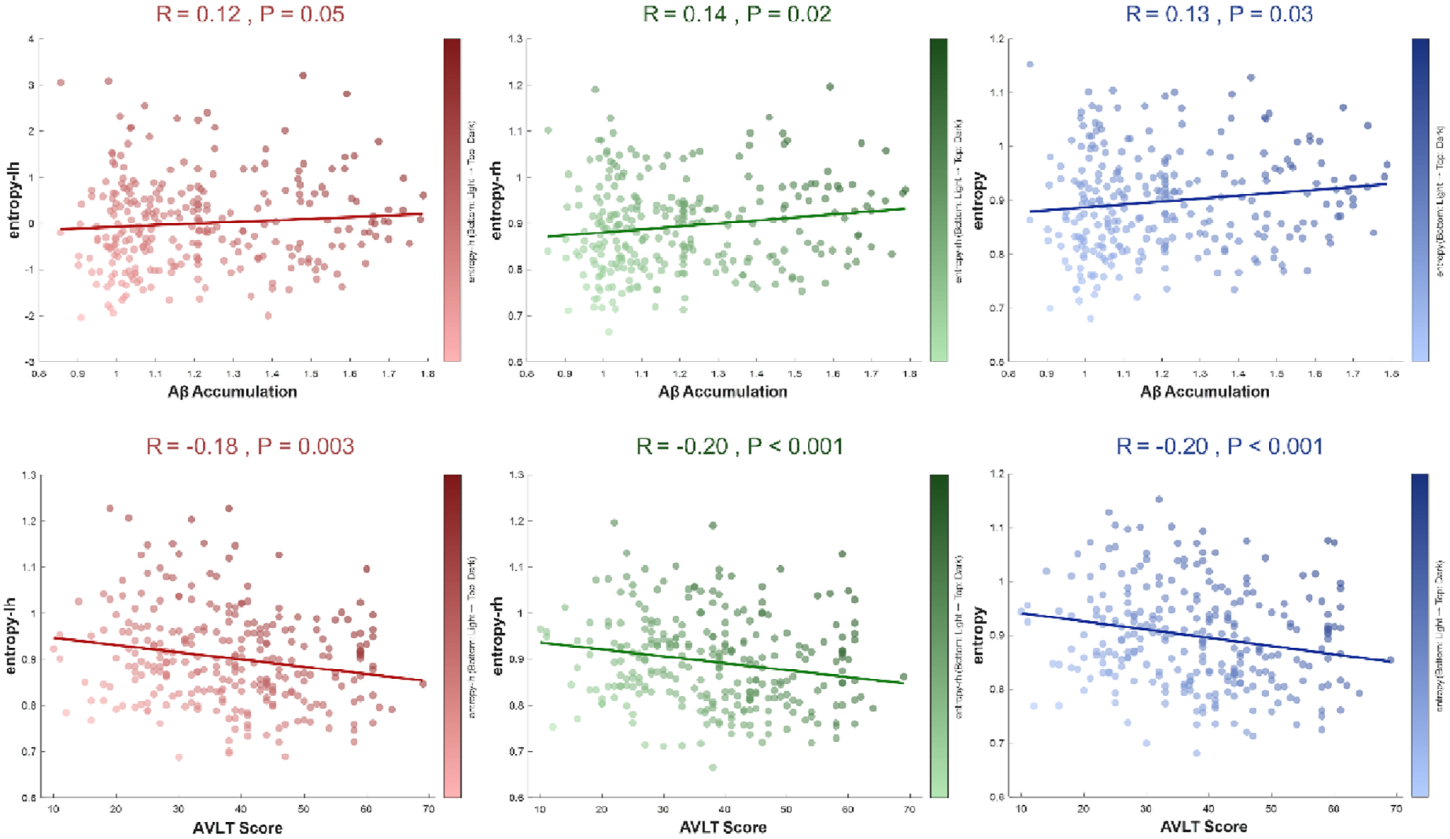
The entropy/temperature index is associated with pathology‐cognition. A) Partial correlations of entropy indexes with Aβ deposition, and AVLT (episodic memory). Entropy significantly correlates with Aβ (r = 0.13–0.14, *p* < 0.05) and AVLT (r = −0.18 to −0.20, *p* < 0.001), but not MMSE. Temperature shows no associations. Sample size: Total N = 410 (BABRI = 135, ADNI = 275).

Entropy indexes further stratified the groups (all *F* > 5.2, *p* < 0.001). Global entropy in the NC group (0.86 ± 0.08) was significantly lower than in MCI‐MCI (0.92 ± 0.10, *p* < 0.001), MCI‐AD (0.92 ± 0.10, *p* < 0.001), and AD groups (0.93 ± 0.11, *p* < 0.001). Right hemisphere entropy displayed a gradient increase: AD (0.94 ± 0.12) > MCI‐AD (0.91 ± 0.10) > NC (0.86 ± 0.09). In contrast, brain temperature parameters showed no significant differences across groups (all *F* < 1.0, *p* > 0.05). Subsequent Spearman correlation analyses (Figure S1, Supporting Information) revealed significant associations between Aβ, entropy indexes, and disease progression (*p* < 0.05). Additionally, partial correlation analyses between entropy/temperature indexes and Aβ deposition, AVLT scores, and MMSE scores revealed that entropy indexes exhibited significant correlations with both Aβ deposition (r = 0.14, *p* < 0.05; r = 0.13, *p* < 0.05) and AVLT scores (r = −0.18, *p* < 0.001; r = −0.20, *p* < 0.001; r = −0.20, *p* < 0.001), but not with MMSE scores (r < 0.1, *p* > 0.05). In contrast, temperature indexes showed no significant associations with Aβ deposition, AVLT scores, or MMSE scores (**Figure** **2**; Table S1, Supporting Information).

The above results indicate that entropy may serve as a robust biomarker for tracking the CN‐to‐AD pathological continuum.

### 2.4 Non‐Linear Entropy Trajectories Link MCI to AD Conversion

**Figure 3:**
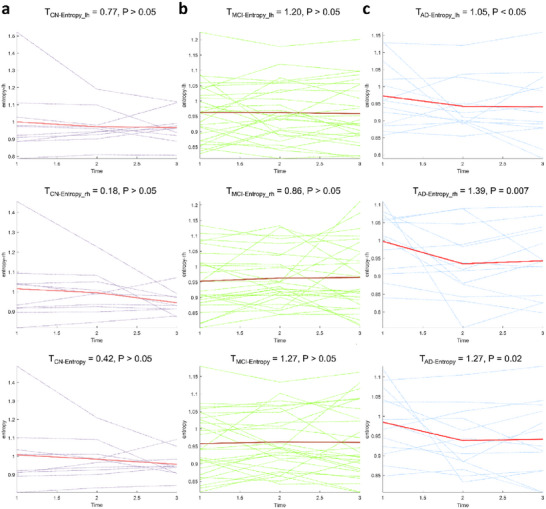
Longitudinal trajectories of entropy across MCI subtypes during cognitive transition: A) Reversed MCI (transitioning to CN) shows linear entropy decline over time, indicating functional normalization; B) Stable MCI maintains consistent entropy levels, reflecting compensatory equilibrium; C) MCI progressors (transitioning to AD) exhibit a biphasic nonlinear trajectory—initial entropy decline (baseline → first follow‐up) followed by plateauing (subsequent follow‐ups) (t = 1.39, *p* < 0.01), marking the transition from compensatory effort to pathological stabilization. Shaded areas denote 95% confidence intervals. Temperature trajectories (not shown) displayed no significant trends across subgroups. Sample sizes: CN (reversed MCI, n = 26); MCI (Stable MCI, n = 64); AD (MCI progressors, n = 35).

We further analyzed longitudinal trends of Shannon entropy and temperature parameters across MCI subgroups during the transition process, as described in Experimental Section (Calculation of Temperature and Shannon's Entropy). Results demonstrated distinct temporal patterns: the reversed MCI exhibited a linear decline in Shannon entropy over time (**Figure** **3**a), while the stable MCI maintained stable entropy levels throughout the observation period (Figure 3b). Notably, only the MCI progressor subgroup displayed a significant non‐linear entropy trajectory during the transition to AD (t  = 1.39, *p* < 0.01). Specifically, entropy declined from baseline to the first follow‐up but plateaued in subsequent follow‐ups (Figure 3c). This biphasic trajectory may reflect the pathological progression of MCI, where initial compensatory mechanisms fail as neurodegeneration accelerates, leading to a stabilization phase marked by advanced pathological burden.

### 2.5 Entropy Predicts Aβ Deposition and Distinguishes Neurodegenerative Stages

The results demonstrated that only Shannon entropy exhibited significant differences across CN, MCI, and AD groups. We therefore constructed linear regression and support vector machine (SVM) models under leave‐one‐out cross‐validation (see Experimental Section). The linear regression model aimed to predict Aβ deposition using entropy indexes, while the SVM model aimed to distinguish normal aging from pathological decline.

**Figure 4:**
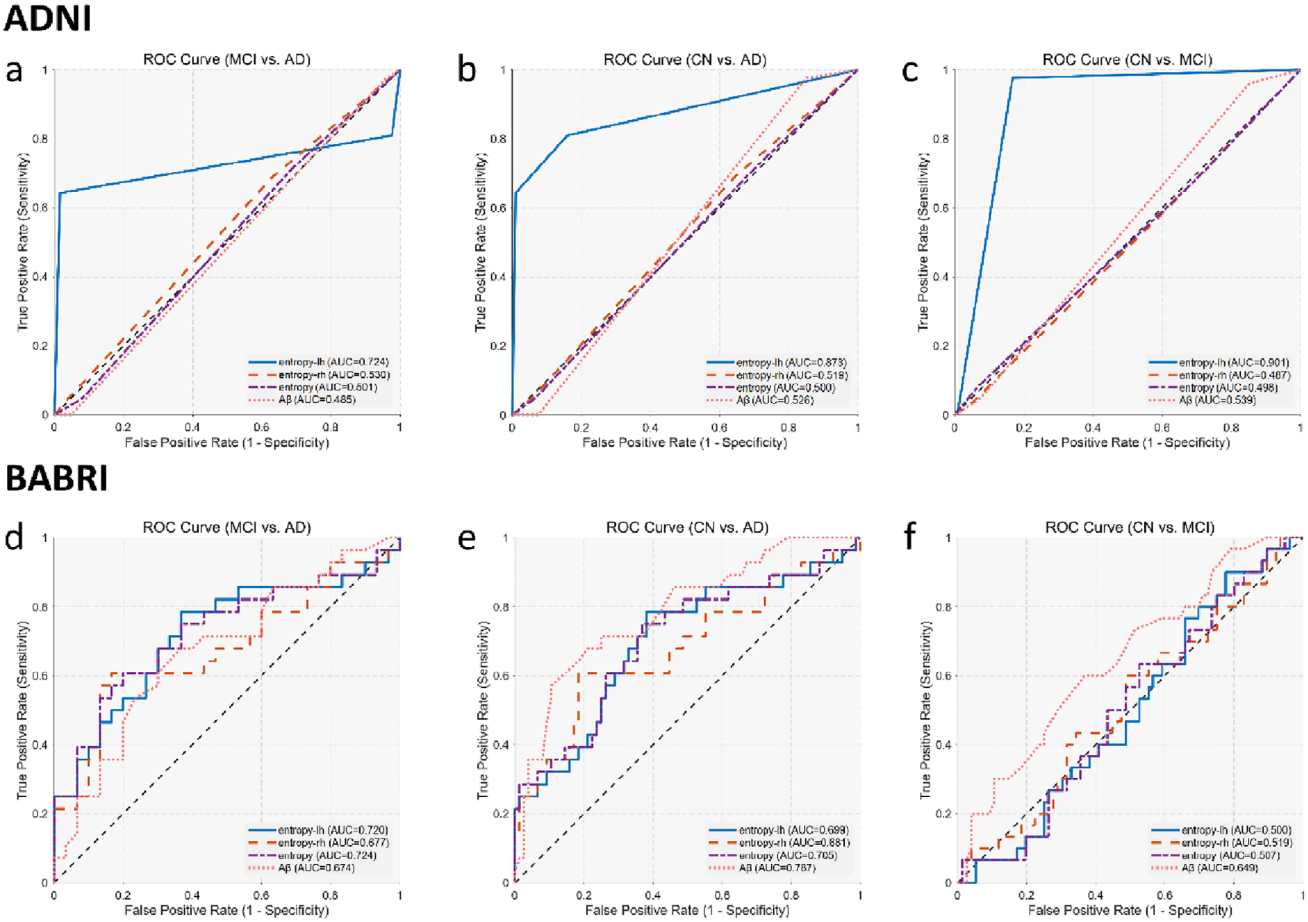
Diagnostic classification performance of entropy indexes in both cohorts across different progression stages. ADNI: a) For MCI versus AD classification, left‐hemisphere entropy (entropy‐lh) achieved the highest performance (AUC = 0.724), outperforming right‐hemisphere entropy (AUC = 0.530), global entropy (AUC = 0.501), and Aβ (AUC = 0.485); b) In CN versus AD classification, entropy‐lh again showed superior discriminative power (AUC = 0.873), while other indexes approached chance level (entropy‐rh = 0.519, global entropy = 0.500, Aβ = 0.526). c) For CN versus MCI classification, entropy‐lh yielded excellent performance (AUC = 0.901), substantially surpassing right‐hemisphere entropy (AUC = 0.487), global entropy (AUC = 0.498), and Aβ (AUC = 0.539). BABRI: d) In MCI versus AD classification, all entropy indexes—left‐hemisphere (AUC = 0.720), right‐hemisphere (AUC = 0.677), and global entropy (AUC = 0.724)—showed comparable performance to Aβ (AUC = 0.674); e) In CN versus AD discrimination, global entropy achieved the best entropy‐based performance (AUC = 0.705), while Aβ outperformed all entropy indexes (AUC = 0.787); f) In CN versus MCI classification, entropy indexes were near chance level (AUCs = 0.500–0.519), whereas Aβ showed modest discrimination (AUC = 0.649).

ROC curve analysis of the SVM classifier revealed the discriminative power of entropy indexes for cognitive impairment subgroups (**Figure** **4**a–c). Left‐hemisphere entropy (entropy‐lh) consistently outperformed right‐hemisphere and global entropy across all classification tasks. Specifically, in CN versus MCI classification, entropy‐lh achieved the highest AUC (0.901), significantly surpassing global entropy (AUC = 0.498) and Aβ (AUC = 0.539). Similarly, in CN versus AD discrimination, entropy‐lh (AUC = 0.873) exceeded right‐hemisphere entropy (AUC = 0.519) and other biomarkers. For MCI versus AD classification, entropy‐lh (AUC = 0.724) also outperformed other biomarkers (all AUCs ≤ 0.501). Notably, Aβ showed limited discriminative capacity across all tasks (AUC = 0.485–0.539), likely due to sample heterogeneity, whereas entropy‐lh maintained robust performance in CN‐MCI (AUC = 0.901) and CN‐AD (AUC = 0.873) classifications, highlighting its potential as a neurodegenerative biomarker.

We also evaluated their diagnostic utility in the BABRI cohort (Figure 4d‐f). In distinguishing MCI from AD (Figure 4d), all three entropy indexes—left‐hemisphere (AUC = 0.720), right‐hemisphere (AUC = 0.677), and global entropy (AUC = 0.724)—demonstrated moderate classification performance, on par with Aβ (AUC = 0.674). This suggests that entropy measures are comparably sensitive to Aβ in capturing disease progression from MCI to AD. In CN versus AD classification (Figure 4e), global entropy achieved the highest entropy‐based AUC (0.705), slightly outperforming left‐hemisphere (AUC = 0.699) and right‐hemisphere (AUC = 0.681). However, Aβ (AUC = 0.787) showed stronger performance in this task, indicating greater separability at the extreme ends of the disease spectrum. In contrast, entropy indexes performed poorly in CN versus MCI discrimination (Figure 4f), with AUCs near chance level (entropy‐lh = 0.500, entropy‐rh = 0.519, global entropy = 0.507), while Aβ showed modest discriminability (AUC = 0.649). These findings suggest that entropy is less sensitive to early‐stage, preclinical changes in this cohort, potentially due to limited signal in structural‐functional disorganization at this early stage.

In generalized linear models predicting Aβ deposition, only right‐hemisphere entropy (RMSE = 0.22, 95% CI [0.21, 0.24]) and global entropy (RMSE = 0.22, 95% CI [0.21, 0.24]) exhibited lower prediction errors compared to the baseline model (RMSE = 0.38, 95% CI [0.35, 0.41]), corresponding to a 42.1% reduction in error (ΔRMSE = −0.16, P_permutation_ < 0.001). Similar trends were observed in the BABRI cohort, where right‐hemisphere entropy (RMSE = 0.13, 95% CI [0.11, 0.14]) and global entropy (RMSE = 0.12, 95% CI [0.11, 0.14]) reduced errors by 38.7% (ΔRMSE = −0.12, P_permutation_ = 0.002) relative to the baseline (RMSE = 0.31, 95% CI [0.27, 0.35]). These findings underscore the dual utility of entropy indexes: left‐hemisphere entropy excels in diagnostic classification, while right‐hemisphere and global entropy robustly predict Aβ burden, collectively positioning entropy as a versatile biomarker in neurodegenerative research.

## 3 Discussion

Through validation in two independent cohorts, this study demonstrates that brain activity entropy (Shannon entropy of gray‐matter eigenmodes) is a valuable new biomarker for Alzheimer's disease (AD). AD patients showed significantly higher entropy (both whole‐brain and hemispheric) than cognitively normal (CN) and mild cognitive impairment (MCI) groups, with left‐hemisphere entropy achieving the highest diagnostic accuracy. This finding underscores entropy's sensitivity in detecting disrupted cortical information integration. Notably, Drachman's 2006 hypothesis that AD is an accelerated form of age‐related entropy increase provides a theoretical foundation.^[^ ^41^ ^]^ Our entropy index quantifies the degree of neural information disorder. In normal aging, entropy increases gradually, reflecting reduced neuronal redundancy and the depletion of cognitive reserve.^[^ ^42^, ^43^ ^]^ However, in the pathological aging associated with AD, entropy increases disproportionately, potentially due to Aβ‐induced ion channel dysfunction that renders the gray matter microenvironment chaotic,^[^ ^44^ ^]^ ultimately disrupting the functional mapping of intrinsic modes.

Moreover, Drachman emphasized a nonlinear relationship between AD neuropathology and cognitive impairment. Our findings suggest entropy reflects the buffering role of cognitive reserve. Under comparable Aβ burden, individuals with lower entropy might delay the onset of dementia through functional reorganization (e.g., compensatory cortical recruitment).^[^ ^45^ ^]^ Conversely, once Aβ exceeds a critical threshold, the brain transitions to pathological aging characterized by the collapse of geometric constraints in gray matter. This breakdown leads to a significant increase in systemic disorder and eventual breakdown of functional integration.^[^ ^46^, ^47^ ^]^

By contrast, the functional reconfiguration temperature index did not differ significantly between diagnostic groups, indicating it mainly captures non‐specific aging effects(like global efficiency declines) rather than AD‐specific changes.^[^ ^48^, ^49^ ^]^ This discrepancy arises from how the two metrics are computed. Entropy is calculated by projecting each region's fMRI time series onto structural eigenmodes of the cortex (Laplacian eigenvectors). This operation yields vertex‐specific weights, and the Shannon entropy of their distribution reflects microscale chaos in neural integration. Importantly, this method preserves the physical constraints imposed by gray matter geometry on local neural dynamics,^[^ ^50^ ^]^ so entropy directly captures AD‐related disorganization at the circuit level. In contrast, the temperature index aggregates signals into large brain regions (e.g., 400 parcels in the Schaefer 2018 atlas), sacrificing fine‐grained variability, and uses eigenmode models of the functional connectivity matrix that capture only linear correlations (ignoring nonlinear dynamics).^[^ ^51^ ^]^ Therefore, entropy is more sensitive to AD‐specific spatiotemporal dynamics at the microscale level,^[^ ^52^ ^]^ whereas temperature, relying on coarse, linear connectivity patterns, tends to lose disease specificity at the macroscale. Consequently, entropy emerges as a superior biomarker for the ultra‐early detection of AD, while temperature may be more appropriate for assessing normative aging processes.

Entropy also reveals heterogeneity within MCI and predicts Aβ deposition. In the ADNI cohort, MCI patients who progressed to AD had significantly higher entropy values and greater Aβ burden than those who remained stable or reverted to normal, supporting entropy's ability to capture the continuum of preclinical pathology. Longitudinally, converters exhibited a nonlinear, biphasic entropy trajectory: an initial compensatory drop in entropy followed by a plateau (decompensation). This dynamic pattern offers a window to anticipate the tipping point of conversion. In the early phase, Aβ plaques accumulate synaptic plasticity and information transfer.^[^ ^53^, ^54^ ^]^ The brain counters with redundant compensatory connectivity, temporarily simplifying network complexity and lowering entropy.^[^ ^55^ ^]^ As the disease progresses, once synaptic loss exceeds ≈30% and gray‐matter geometry is severely disrupted, compensatory mechanisms collapse. Entropy then plateaus at an elevated level as widespread neurodegeneration leads to functional failure.^[^ ^56^ ^]^

Left‐hemispheric entropy played a dominant role in diagnostic classification, achieving an AUC of 0.901 for distinguishing CN versus AD. This likely reflects the particular vulnerability of left hemisphere regions, such as default mode network hubs involved in language and logical integration, to AD pathology.^[^ ^57^ ^]^ Elevated entropy in these areas indicates structural damage and functional decline, potentially flagging prodromal AD signs earlier than traditional markers. Right‐hemispheric entropy, on the other hand, correlated more with molecular pathology. The right hemisphere supports spatial navigation and episodic memory, functions often affected early in AD.^[^ ^42^ ^]^ Notably, the right posterior cingulate cortex, an early site of Aβ accumulation, showed increased entropy, suggesting impaired clearance mechanisms, possibly involving the glymphatic system.^[^ ^58^, ^59^ ^]^ Thus, right‐hemisphere entropy may serve as a sensitive marker of early amyloid pathology even before cognitive symptoms appear.

A key advantage of entropy over gray‐matter eigenmodes is that it can be computed directly from routine MRI, combining structural T1 with a short resting‐state fMRI, which makes it highly suitable for clinical use. Our findings suggest several applications: first, elevated left‐hemisphere entropy in patients with cognitive complaints—relative to age‐ and sex‐adjusted norms—may help identify individuals at higher risk for AD and warrant closer monitoring. Second, since global or right‐hemisphere entropy correlates with Aβ burden, patients with higher entropy percentiles can be prioritized for confirmatory biomarker tests such as amyloid PET or CSF when resources are constrained. Finally, entropy provides a compact index to track disease trajectory in MCI patients, where longitudinal changes (Δ‐entropy), reported alongside cognitive scores, serve as interpretable markers to distinguish progression from stability over time. For successful clinical deployment, the study recommends standardized data preprocessing and quality control, reporting of entropy values in age/sex‐adjusted percentile bands (e.g., low/average/high in radiology reports), and consistent statistical reporting (with proper multiple‐comparison corrections and effect sizes) in research studies. Implementing these steps would position entropy as a non‐invasive adjunct to routine imaging—informing referral decisions, test prioritization, and follow‐up scheduling—while **complementing** (not replacing) clinical evaluations and established fluid/PET biomarkers.

## 4 Limitation

Despite the strong discriminative power of entropy indexes demonstrated across cohorts, several limitations should be acknowledged, each accompanied by a concrete mitigation step.

First, pathological validation remains incomplete. Although entropy showed a modest correlation with Aβ‐PET (r = 0.14), tau‐PET and cerebrospinal fluid (CSF) biomarkers were not included. Since tau pathology is more closely linked to cognitive decline than Aβ, future studies should assess whether entropy also captures tau‐related network disintegration.

Second, MCI subtyping was based solely on clinical progression (MCI‐CN, MCI‐MCI, MCI‐AD), without biomarker‐defined classes (e.g., A+T+ vs A−T−). This may obscure distinct entropy trajectories across biologically diverse MCI subtypes. We will jointly subtype MCI using clinical course and AT(N) status, then re‐estimate entropy trajectories with mixed‐effects models by subtype.

Third, the lack of significant group differences in the temperature index (all *p* > 0.05) may reflect methodological constraints: parcel‐level smoothing can dilute vertex‐level dynamics. We will evaluate vertex‐wise temperature and spectral/diffusion‐based formulations (multi‐scale diffusion times t), and repeat analyses with finer parcellations.

Fourth, the biphasic entropy trajectory in MCI converters relied on limited follow‐up. We will design denser longitudinal sampling (e.g., 6–12‐month intervals) and fit mixed‐effects splines with subject‐specific knots to estimate the inflection point more precisely.

Fifth, the relationship between entropy and cognition appears domain‐specific: it correlates with episodic memory (AVLT) but not global cognition (MMSE). We will expand the cognitive battery to include executive and visuospatial measures and derive latent domain scores (composites) to reduce noise.

## 5 Conclusion

This multi‐cohort study establishes Shannon entropy as a robust, dual‐purpose biomarker for Alzheimer's disease (AD) pathology, demonstrating significant efficacy in both diagnostic classification and pathological prediction. Entropy index consistently differentiated AD from NC and MCI across BABRI and ADNI cohorts, with left‐hemisphere entropy being the optimal classifier for neurodegenerative staging and right‐hemisphere entropy dominating Aβ burden prediction. Moreover, entropy was able to stratify clinically defined MCI into biologically distinct subtypes and revealed a biphasic entropy shift in MCI→AD converters. The hemispheric asymmetry in entropy utility offers a unified framework for “structure‐function decoupling” in association cortices. Entropy's superiority over Aβ‐PET and resistance to demographic confounds make it a cost‐effective, non‐invasive tool for early risk stratification and identifying the “inflection point” in MCI biphasic trajectories for precision neuromodulation.

We establish **Shannon entropy of gray‐matter eigenmodes** as a compact, interpretable biomarker that (i) differentiates CN/MCI/AD with **best AUC = 0.901 (CN** vs **MCI, left‐hemisphere)**, (ii) predicts Aβ burden (right/global entropy), and (iii) stratifies MCI subtypes with a **biphasic progression trajectory** in converters, across two independent cohorts (BABRI, ADNI). These findings support entropy as a non‐invasive index of cortical disorganization with practical clinical potential for **early risk stratification** and **progression monitoring**. Limitations include biomarker coverage (no tau‐PET/CSF), sensitivity to eigenmode/parcellation choices, and cross‐site variability. Future work will prioritize prospective, multi‐site validation; integration with tau and fluid biomarkers; harmonization frameworks; and exploring vertex‐wise “temperature” variants to complement entropy.

## 6 Experimental Section

### Participants

The participants were from the Beijing Aging Brain Rejuvenation Initiative (BABRI)^[^ ^60^ ^]^ and the Alzheimer's Disease Neuroimaging Initiative (ADNI).^[^ ^61^ ^]^ All participants were classified as NC or MCI, or AD at baseline. All the participants completed florbetapir PET, structural MRI, and resting functional MRI. Briefly, the diagnostic criteria for MCI included subjective memory complaints, impairment in at least one cognitive domain (1.5 standard deviations or more), relatively preserved general cognitive function, and intact ability to perform activities of daily living.^[^ ^62^ ^]^ The criteria for normal cognition were no cognitive complaints, a Mini‐Mental State Examination (MMSE) score of no less than 24, and being able to perform the normal activities of daily life. AD was diagnosed according to the criteria of the National Institute of Neurological and Communicative Disorders and Stroke and the Alzheimer's Disease and Related Disorders Association Dementia, and further evaluated by brain CT or MRI.^[^ ^63^, ^64^ ^]^ A total of 135 eligible participants from the BABRI and a total of 275 eligible participants from the ADNI were included in this study.

In the data derived from ADNI, we also incorporated the longitudinal follow‐up information of the MCI group. All participants in the MCI follow‐up cohort underwent structural MRI and resting‐state functional MRI examinations. A total of 49 eligible participants were ultimately enrolled. Among them, 10 subjects were classified as reversed MCI (reverting from MCI to cognitively normal (CN) during follow‐up), 26 maintained stable MCI diagnoses, and 13 progressed to Alzheimer's disease (AD) (transitioning from MCI to AD).

The study was conducted in accordance with the institutional review board (IRB) at the Imaging Center for Brain Research at Beijing Normal University (protocol code ICBIR_A_0041_002_02 and date of approval 03.2015). All participants provided written informed consent for our protocol, which was approved by the ethics committee of the State Key Laboratory of Cognitive Neuroscience and Learning, Beijing Normal University.

### MRI Data Acquisition and Processing

High‐resolution T1‐weighted MRI data were collected from each participant from the BABRI and ADNI using either 1.5‐T scanners (participants from ADNI‐1) or 3‐T scanners (participants from BABRI and ADNI‐GO&2); the acquisition parameters for each study have been published previously.^[^ ^65^, ^66^ ^]^

### MRI Image Acquisition and Data Processing

For all subjects, cortical reconstruction of T1‐weighted images was performed using FreeSurfer version 5.3 (http://surf er.nmr.mgh.harvard.edu).^[^ ^67^ ^]^ This process involved registration to a template, intensity normalization, gray/white matter segmentation, and tessellation of gray/CSF and white/gray boundaries. Cortical surfaces were inflated and normalized via spherical registration, with cortical thickness defined as the shortest distance between the pial and white matter surfaces. The average regional cortical thickness was calculated without manual correction. The procedure included bias field correction, intensity normalization, and skull stripping using a watershed algorithm, followed by white matter segmentation, surface definition, and topology correction of the reconstructed surfaces. We used a triangular surface mesh representation of the mid‐thickness human cortical surface. This representation, comprising 32 492 vertices in each hemisphere, was obtained from a down‐sampled, left–right symmetric version of the FreeSurfer's fsaverage population‐averaged template (https://github.com/ThomasYeoLab/CBIG/tree/master/data/templates/surface/fs_LR_164k). Note that the template is independent of the data sample used in our analyses, thus avoiding concerns about circularity.

### The Preprocessing Pipeline for Functional Neuroimaging Data

Resting‐state fMRI data were preprocessed using fMRIPrep (https://fmriprep.org/, V20.1.3), a Python‐based automated tool integrating advanced neuroimaging packages like FSL, ANTs, FreeSurfer, and AFNI, designed for optimized fMRI preprocessing.^[^ ^68^ ^]^

The procedure included head motion estimation, slice ‐ timing correction, fMRI‐to‐T1w registration, fMRI‐to‐MNI152 standard ‐ space normalization, confound estimation, and regression. Specifically, head motion parameters were estimated and saved for subsequent regression. Slice‐timing correction adjusted for differences in slice acquisition times. The fMRI data were coregistered to the T1 ‐ weighted structural image for precise alignment and then normalized to the MNI152 space for group‐level analysis. Confounds such as head motion parameters, white matter, and CSF signals were estimated for regression. Post ‐ preprocessing steps comprised linear drift removal, Gaussian smoothing (6 mm FWHM), and nonlinear band‐pass filtering (0.01–0.1 Hz). Quality control involved visually checking T1w images and excluding subjects with excessive head motion (maximum head motion > 3 mm or 3°, or mean FD > 0.5 mm). All analyses were conducted at the voxel level to ensure accuracy and consistency.

### Spatiotemporal Mode Decomposition of Neural Dynamics Through Structural Eigenmodes

The computational pipeline for investigating the coupling between cortical geometry and functional activity was implemented through four sequential modules, with all analyses conducted in individual cortical space to preserve neuroanatomical specificity.

**Structural Eigenmode Computation**: Cortical surface reconstruction was performed using FreeSurfer v7.2 (recon‐all pipeline). Laplace‐Beltrami Operator (LBO) was defined based on the metric describing the mid‐thickness surfaces geometry. Specifically, Mid‐thickness surfaces were converted to VTK format, and the LBO was given through custom Python scripts.^[^ ^30^ ^]^ Eigenmodes were computed using the eigenvalue equation:

(1) $$\Delta \psi_{i}=-\lambda_{i}\psi_{i}$$

where Δ denotes the LBO, ψ_*i*_ represents the *i*‐th eigenmode, and λ_*i*_ is the corresponding eigenvalue. This computation was generating frequency‐ranked cortical vibration modes.

**Functional Data Processing**: First, FreeSurfer's mri_vol2surf (v7.2) implemented grayordinate‐wise projection through ray‐casting sampling (256 iterations, 0.1 mm step size) onto the native space, preserving cortical columnar organization. Second, vertex‐level temporal dynamics were encoded in sparse matrices (N = vertices × M TRs), which maintained native temporal resolution. This processing chain ensured topological correspondence between structural eigenmodes and functional time series across spatial scales.

**Cortical Mask Generation**: Individual‐specific cortical masks were generated through a three‐stage surface‐constrained protocol. First, probabilistic tissue segmentation from FreeSurfer's aparc.aseg.mgz output was thresholded at a 50% confidence level to minimize partial volume effects. Subsequently, a binarization procedure assigned value 1 to vertices within anatomically verified cortical regions (Desikan‐Killiany atlas) and 0 to non‐cortical structures (subcortical nuclei, white matter, ventricles). Finally, the volumetric mask was projected onto native cortical surfaces, generating exclusion masks that systematically removed subcortical and ventricular components while preserving cortical geometry in individual surface space. This process ensured topological consistency between structural and functional data representations.

**Computational Implementation**: All pipelines were executed on HCP‐style processing clusters using Python 3.8 and Bash scripting. Surface operations employed FreeSurfer v 5.3 and Connectome Workbench v1.5 commands, with quality assurance via visual inspection of surface‐normal vectors and Euler characteristics.

### Calculation of Temperature and Shannon's Entropy

Cortical eigenmodes (200 modes) and resting‐state fMRI time series (N vertices × M TRs) were first aligned in individual surface space using FreeSurfer‐processed mid‐thickness meshes. Shannon's entropy H was computed via:^[^ ^69^ ^]^

(2) $$H=-\sum_{i=1}^{200}\left(\beta_{i}^{2}log\beta_{i}^{2}\right)$$

where β_*i*_ were obtained by projecting the time‐averaged fMRI data onto each eigenmode basis function. Temperature estimation is given by a fitting of the functional connectivity (FC) reconstruction accuracy:

(3) $$R^{2}\left(i\right)=1-a\cdot e^{-b\cdot i}$$

where *R* ^2^(*i*) represents the cumulative reconstruction accuracy of functional connectivity (FC) using the first i structural eigenmodes and *R* ^2^(*i*) was fitted to a cumulative distribution function of an exponential distribution. The characteristic temperature parameter *T* was defined as T≔1/b through nonlinear least‐squares optimization (MATLAB fminsearch, initial guess [a = 1, b = 0], convergence tolerance 1^e‐4^). FC matrices were parcellated using the Schaefer‐400 atlas,^[^ ^70^ ^]^ with upper‐triangular elements correlated against empirical FC to compute reconstruction accuracy. All computations were constrained to cortical vertices using individually masked surfaces, ensuring exclusion of subcortical artifacts.

### Computational Complexity and Resources

We use Big‐O *O*(·) to denote asymptotic upper bounds (constants and lower‐order terms omitted). *nnz*(*L*) denotes the number of non‐zero entries in the sparse matrix *L* (“number of non‐zeros”). We also use *V* as cortical vertices, *k* the retained eigenmodes, *M* fMRI timepoints, and *P* the number of parcels (Schaefer‐400).

**Eigenmodes**: Computing the top‐*k* eigenpairs of the sparse Laplace‐Beltrami operator *L* on cortical surfaces (per subject) with an implicitly restarted Lanczos/ARPACK‐style solver runs in *O*(*k* *nnz*(*L*)) time and *O*(*nnz*(*L*) + *kV*) memory (V: vertices).

**Projection & entropy :** Projecting fMRI time‐series (V × M) onto eigenmodes to obtain coefficients ${\left\{a_{i}\right\}}_{i=1}^{k}$ is O(VM); computing Shannon entropy from{a_i_}is *O*(*k*).

**Temperature**: Parcel‐wise FC estimation with *P* parcels (Schaefer‐400) is *O*(*P* ^2^ *M*); cumulative reconstruction across*i* = 1.*k* modes and exponential‐decay fitting is *O*(*k* *P* ^2^).

**Learning**: Classification/regression uses only a handful of features (entropy_lh/rh/global ± Aβ), so training cost is negligible relative to eigenmode and FC steps.

**Pipelines**: All computations are integrated with fMRIPrep‐based preprocessing and FreeSurfer surfaces as already described.

### Statistical Analysis

To compare demographic characteristics (analyzed via chi‐square test for sex distribution), temperature, and entropy indexes across groups, an analysis of covariance (ANCOVA) was performed with age, education, and sex as covariates. Partial correlation analyses were conducted to assess the relationship between Aβ accumulation and entropy indexes across all participants, adjusting for the aforementioned covariates. Spearman correlation was employed to evaluate associations between disease progression stages and both entropy and temperature indexes.

**Longitudinal Rate of Change Analysis**: For each participant, the rate of entropy change between consecutive follow‐ups was calculated as (Entropy_t+1_–Entropy_t_)/Time. Paired *t*‐tests were conducted to compare these rates across the two intervals within each subgroup (reversed MCI, stable MCI, and progressed MCI), aiming to identify non‐linear trends in entropy and temperature dynamics during disease progression. Differences are denoted with the Greek delta (Δ).

**Multi‐Class SVM Classification**: A multi‐class support vector machine (SVM) model with a “one‐versus‐all” strategy was implemented to evaluate the classification efficacy of neuroimaging indexes (left‐hemisphere entropy, right‐hemisphere entropy, global entropy, and Aβ deposition) across cognitive states (CN, MCI, AD). Leave‐one‐out cross‐validation (LOOCV) was employed: for each subgroup comparison (CN vs MCI, CN vs AD, MCI vs AD), one sample was iteratively excluded as the test set, while the remaining samples were used to train the model. Model performance was assessed via sensitivity, specificity, and AUC values, with emphasis on hemispheric entropy asymmetry and Aβ pathology in predicting early‐stage transitions.

**Prediction of Aβ Deposition**: Under the LOOCV framework, linear regression models were constructed to predict standardized Aβ deposition values using individual entropy indexes as predictors. For each indexes, a generalized linear regression model was trained on all but one sample, and the root mean square error (RMSE) was computed to evaluate prediction accuracy. A baseline model predicting Aβ using the sample mean was established, and effective predictors were identified as those with RMSE significantly lower than the baseline (ΔRMSE < 0, *P*_permutation_ < 0.05).

**Multiple‐comparison control**: To control type‐I error, we used **Bonferroni correction (α = 0.05)** within predefined families of tests in each cohort. For group analyses (ANCOVA), families comprised all post‐hoc pairwise contrasts among diagnostic groups and the three entropy indices (left, right, global). For association analyses, families comprised all tests across indices and outcomes (e.g., Aβ and AVLT). For longitudinal analyses in MCI subgroups, families comprised tests across subgroups and indices (and intervals where applicable). For predictive analyses (Aβ regression under LOOCV), *p*‐values were Bonferroni‐adjusted across entropy‐index predictors. Where applicable, we report adjusted *p*‐values as *p*<sub>Bonf</sub>; all tests are two‐sided.

**Post hoc Sensitivity Analysis**: Because this study is retrospective with fixed cohort sizes, an a priori power calculation was not applicable. Instead, we conducted a post hoc sensitivity analysis to estimate the minimal detectable effect sizes (MDE; Cohen's d) for the main group comparisons at α = 0.05 and 1 − β = 0.80. For the ADNI cohort, the MDEs were d ≈ 0.37 for CN (n = 107) versus MCI (n = 126), d ≈ 0.50 for MCI (n = 126) versus AD (n = 42), and d ≈ 0.45 for CN (n = 107) versus AD (n = 42). For the BABRI cohort, the MDEs were d ≈ 0.61 for CN (n = 76) versus MCI (n = 30), d ≈ 0.72 for MCI (n = 30) versus AD (n = 29), and d ≈ 0.62 for CN (n = 76) versus AD (n = 29).

## Conflict of Interest

The authors declare no conflict of interest.

## Author Contributions

Y.L. and G.L. contributed equally to this work. Y.L. and G.L. developed overall methodology and carried out the theoretical analysis. Y.L., G.L., X.Z., K.C., and X.L. participated in data analysis. Y.L., G.L., and X.L. contributed to the manuscript preparation and revision. Z.Z. supervised the work team.

## Supporting information



Supporting Information

## Data Availability

The data sets analyzed during the study are available from the corresponding author upon reasonable request.
