# Supplementary material for: Shannon Entropy of Gray Matter Eigenmodes: A Novel Biomarker for Alzheimer's Disease and Heterogeneous MCI Trajectories
Source: Adv Sci (Weinh). 2025 Nov 4;13(1):e11614. doi: 10.1002/advs.202511614 (PMC12767045; doi:10.1002/advs.202511614)
Supplement: Supplementary file 1 — Supporting Information [file ADVS-13-e11614-s001.docx]

**
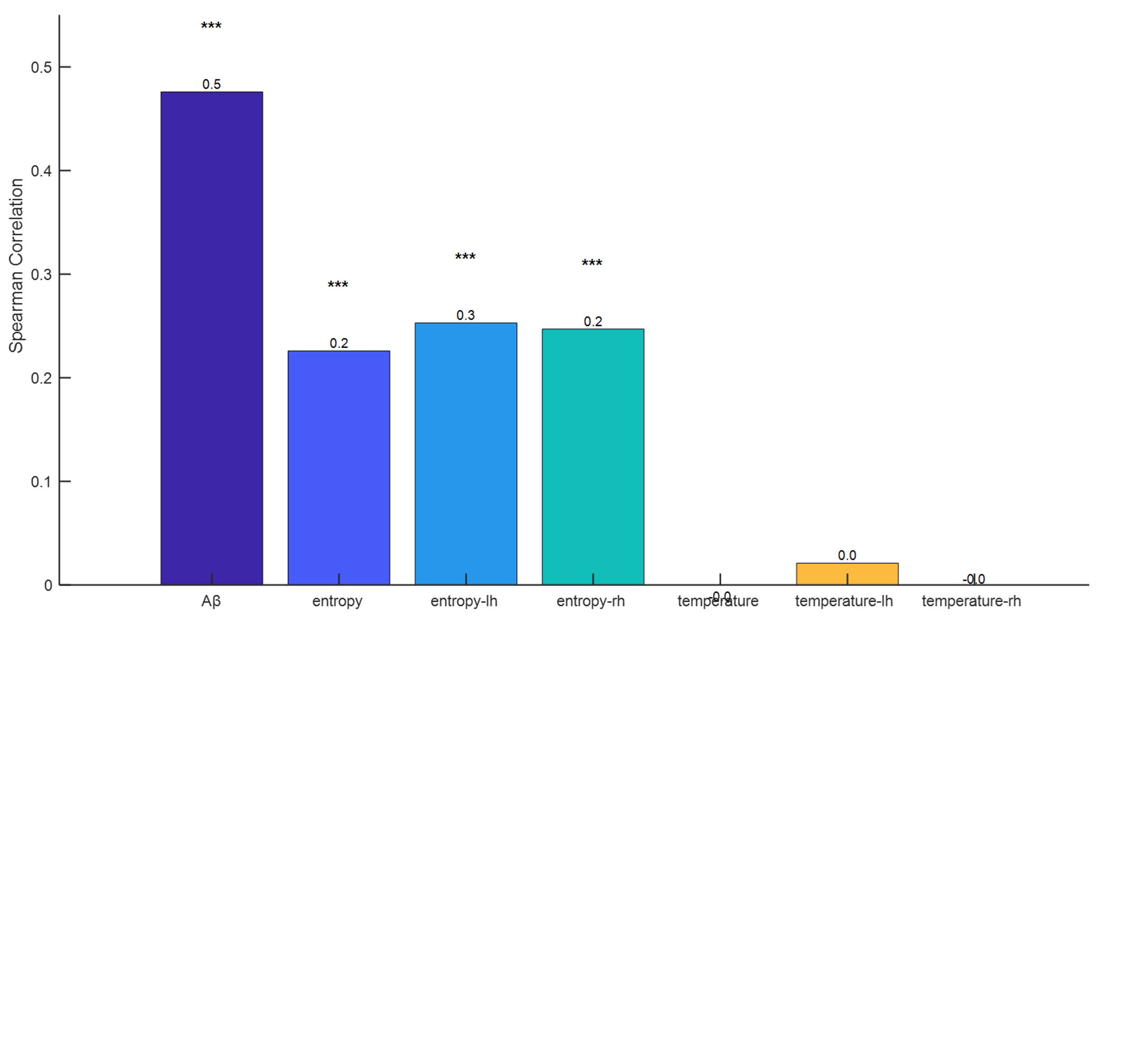
**

**Supplementary Figure S1. The cross-group correlation between entropy and Aβ deposition.** Spearman correlations between Aβ deposition and entropy metrics across cognitive transition groups (NC, MCI-CN, MCI-MCI, MCI-AD, AD). Temperature metrics display no significant trends. **Sample size:** Total N = 410 (BABRI = 135, ADNI = 275).

**
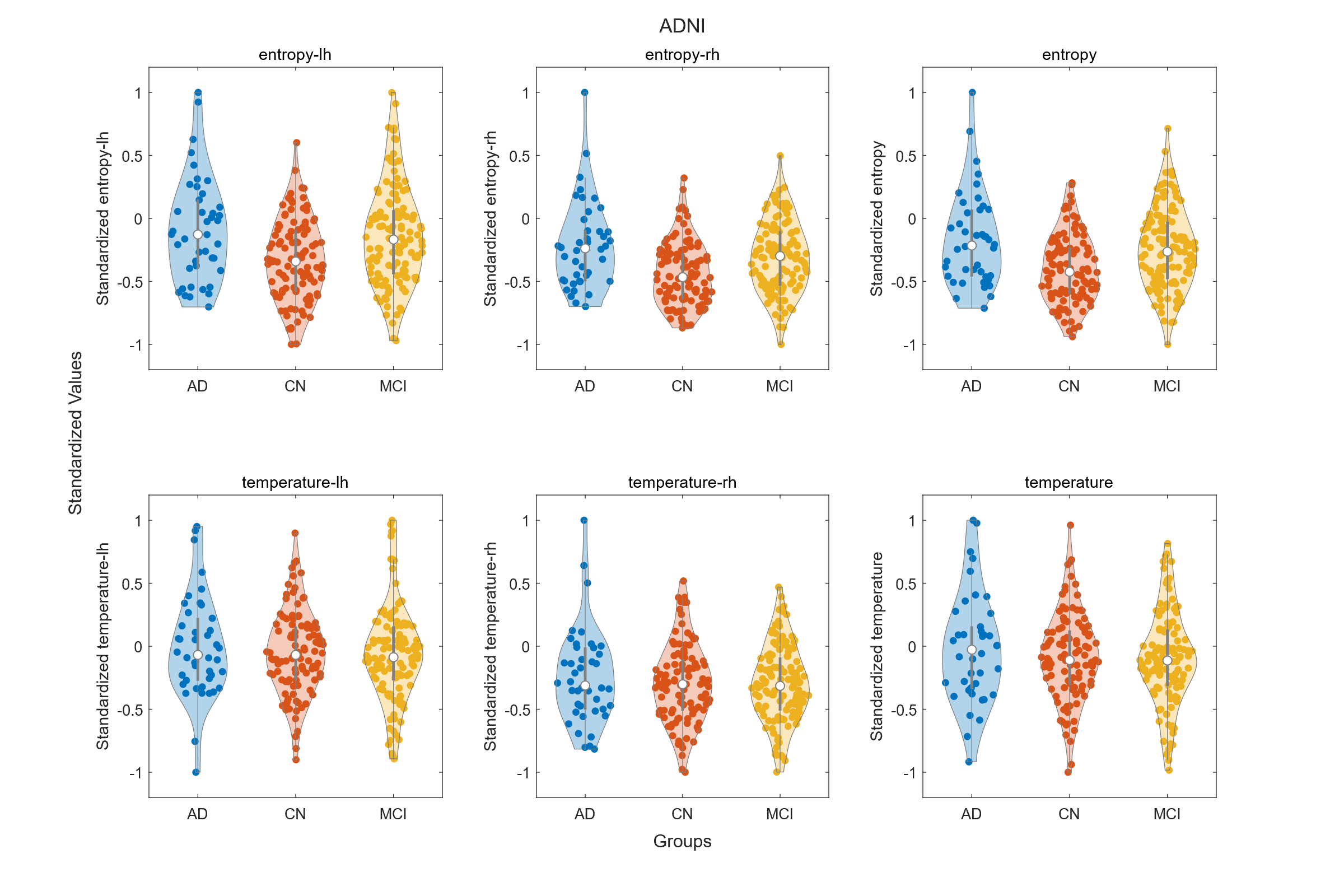
**

**Supplementary Figure S2** **ADNI: Violin Plots of Standardized Entropy and Temperature Across Diagnostic Groups**
Violin plots show the distributions for entropy-lh, entropy-rh, entropy, temperature-lh, temperature-rh, and temperature; colors denote diagnostic groups (AD = blue, CN = orange, MCI = yellow); violin width encodes kernel density; the white dot marks the median; the thick bar indicates the interquartile range (IQR); the thin line shows the data range (min–max); overlaid jittered dots represent individual participants; the y-axis shows standardized values (z-scores). **Sample sizes:** ADNI cohort: total = 275 (NC = 107, MCI = 126, AD = 42).

**
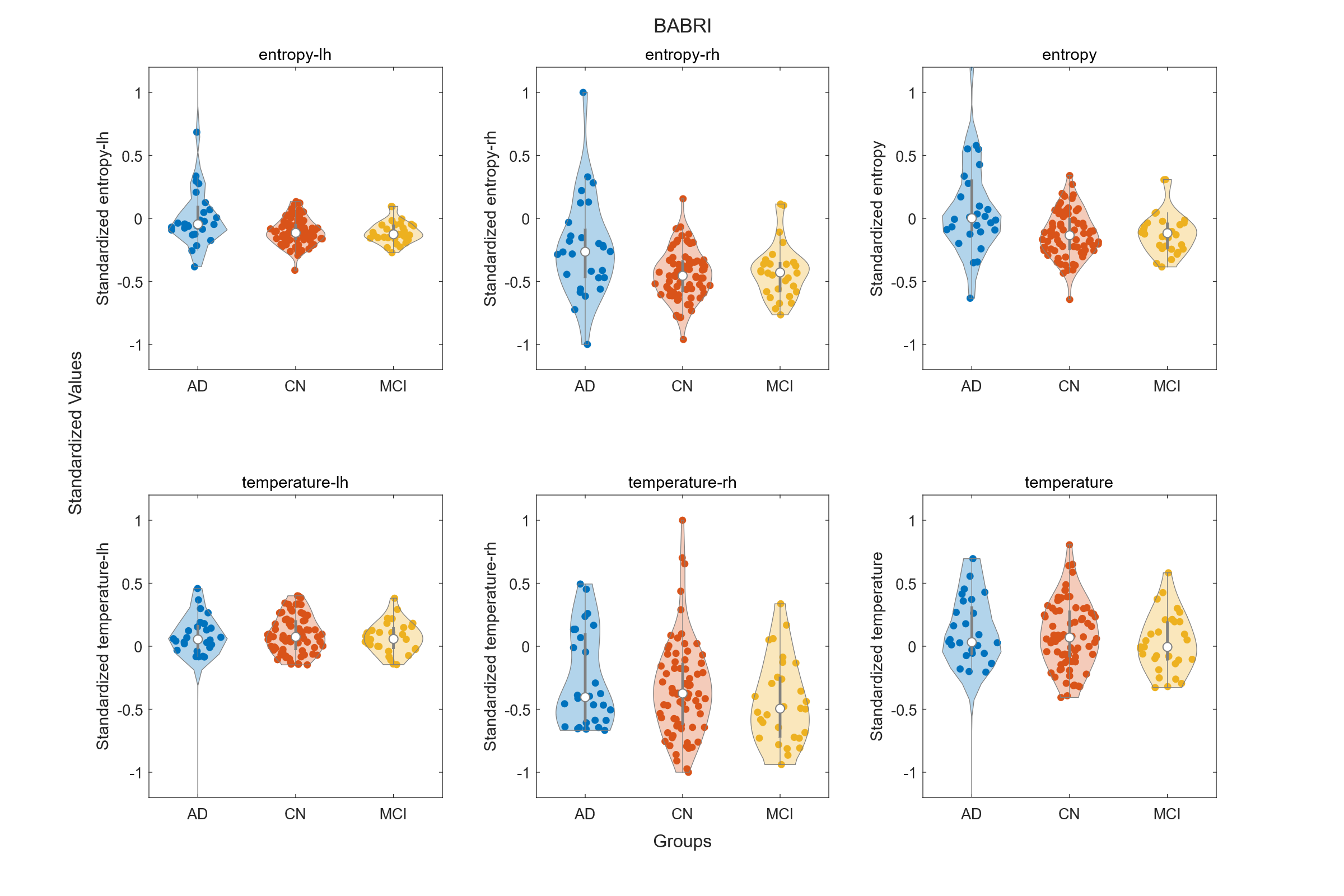
**

**Supplementary Figure S3** **BABRI: Violin Plots of Standardized Entropy and Temperature Across Diagnostic Groups**
Violin plots show the distributions for entropy-lh, entropy-rh, entropy, temperature-lh, temperature-rh, and temperature; colors denote diagnostic groups (AD = blue, CN = orange, MCI = yellow); violin width encodes kernel density; the white dot marks the median; the thick bar indicates the interquartile range (IQR); the thin line shows the data range (min–max); overlaid jittered dots represent individual participants; the y-axis shows standardized values (z-scores). **Sample sizes:** BABRI cohort: total = 135 (NC = 76, MCI = 30, AD = 29).

**Supplementary Table S1** Correlation of entropy and temperature with Aβ accumulation and cognitive performance

|  | e_lh | e_rh | entropy | t_lh | t_rh | temper | Total | MMSE | AVLT |
| --- | --- | --- | --- | --- | --- | --- | --- | --- | --- |
| e_lh | -- | 0.85^***^ | 0.96^***^ | 0.12 | 0.08 | 0.10 | 0.08 | -0.04 | -0.18^***^ |
| e_rh | 0.85^***^ | -- | 0.96^***^ | 0.03 | 0.00 | 0.02 | 0.14^*^ | -0.08 | -0.20^***^ |
| entropy | 0.96^***^ | 0.96^***^ | -- | 0.08 | 0.04 | 0.06 | 0.13^*^ | -0.06 | -0.20^***^ |
| t_lh | 0.12 | 0.03 | 0.08 | -- | 0.68^***^ | 0.91^***^ | 0.05 | -0.05 | -0.06 |
| t_rh | 0.08 | 0.00 | 0.04 | 0.68^***^ | -- | 0.93^***^ | 0.05 | -0.07 | -0.09 |
| temper | 0.10 | 0.02 | 0.06 | 0.91^***^ | 0.93^***^ | -- | 0.05 | -0.07 | -0.08 |
| Aβ | 0.12 | 0.14^*^ | 0.13^*^ | 0.05 | 0.05 | 0.05 | -- | -0.39^***^ | -0.41^***^ |
| MMSE | -0.04 | -0.08 | -0.06 | -0.05 | -0.07 | -0.07 | -0.39^***^ | -- | 0.59^***^ |
| AVLT | -0.18^***^ | -0.20^***^ | -0.20^***^ | -0.06 | -0.09 | -0.08 | -0.41^***^ | 0.59^***^ | -- |

Note: Pearson correlation coefficients (*r*) are reported; *p*-values in parentheses: ** < .05,* ******** *< .01, ***** < .001*; Diagonal entries = 1.00 (variable self-correlation); Bonferroni correction applied for multiple comparisons

**Supplementary Table S2** Dataset characteristics and analytic features

| **Cohort** | **Total N** | **Group counts (CN / MCI / AD)** | **Longitudinal MCI subtypes (ADNI)** | **Modalities** | **Key analytic features** |
| --- | --- | --- | --- | --- | --- |
| BABRI | 135 | 76 / 30 / 29 | — | T1-MRI, rs-fMRI, Aβ-PET; 3 T (BABRI) | entropy_lh, entropy_rh, entropy_global; temperature_lh, temperature_rh, temperature_global; Aβ |
| ADNI | 275 | 107 / 126 / 42 | 49 MCI with follow-up: 10 reverted (MCI→CN), 26 stable, 13 progressed (MCI→AD) | T1-MRI, rs-fMRI, Aβ-PET; 1.5 T/3 T (ADNI) | same as above |
